# J.A. Schumpeter and T.B. Veblen on economic evolution: the dichotomy between statics and dynamics

**DOI:** 10.1080/09672567.2015.1018294

**Published:** 2015-04-08

**Authors:** Marlies Schütz, Andreas Rainer

**Keywords:** T.B. Veblen, J.A. Schumpeter, dynamic economic systems, pure economics, evolutionary economics

## Abstract

At present, the discussion on the dichotomy between statics and dynamics is resolved by concentrating on its mathematical meaning. Yet, a simple formalisation masks the underlying methodological discussion. Overcoming this limitation, the paper discusses Schumpeter's and Veblen's viewpoint on dynamic economic systems as systems generating change from within. It contributes to an understanding on their ideas of how economics could become an evolutionary science and on their contributions to elaborate an evolutionary economics. It confronts Schumpeter's with Veblen's perspective on evolutionary economics and provides insight into their evolutionary economic theorising by discussing their ideas on the evolution of capitalism.

## 1. Introduction

Fritz Machlup in his 1959 article on *Statics and Dynamics: Kaleidoscopic Words* listed 43 different definitions on the separation of ‘statics’ and ‘dynamics’, brought forth by 37 economists at his time and before him. Against the ongoing discussions of how these terms were understood and how the respective economic theories ought to be assessed, he stressed, first, that rigorous definitions of statics and dynamics were still lacking, and second, that economists had used the distinction between the two terms to differentiate their own work from the work of their contemporaries and predecessors:

For more than twenty years I have been telling my students that one of the widespread uses of ‘Statics’ and ‘Dynamics’ was to distinguish a writer's own work from that of his opponents against whom he tried to argue. Typically, ‘Statics’ was what those benighted opponents have been writing; ‘Dynamics’ was one's own, vastly superior theory. (Machlup 1959, p. 100)

Machlup then suggested circumventing the use of the terms ‘statics’ and ‘dynamics’ whenever possible to avoid misunderstandings. Instead, these terms ought to be replaced by descriptions like growth theory, theory of the evolution of economic institutions, time-series analysis, trend analysis, sequence analysis, period analysis, and so on, such that ‘more often than not we should be able to do without the terms Statics and Dynamics’ (1959, p. 110). Some years before Machlup, Ragnar Frisch did not abandon the use of ‘statics’ and ‘dynamics’ but tried to clarify their meaning. In his 1930 Yale lectures he noted that ‘the distinction between statics and dynamics is a *distinction between two different ways of thinking, not a distinction between two different kinds of phenomena*’ (Frisch 2011, p. 44). A dynamic analysis was understood by Frisch as one which ‘tries to explain *how one situation grows out of the preceding*’ (2011, p. 44). Already before Machlup and Frisch, the meanings of the terms ‘statics’ and ‘dynamics’ were scrutinised by the young Joseph Alois Schumpeter and Thorstein Bunde Veblen, independently of each other. Their discussions were also related to the concepts of ‘development’ and ‘evolution’.

Schumpeter in *Das Wesen und der Hauptinhalt der theoretischen Nationalökonomie* (1970[1908], henceforth *Das Wesen*) took pains to highlight this aspect of economic theory:

My exposition is based on the fundamental distinction between economic ‘statics’ and ‘dynamics’, an aspect of utmost importance. […] ‘Dynamics’ differs in every respect from ‘statics’, methodologically as well as in terms of content. (*Das Wesen*, p. XIX)

For Veblen ‘dynamics’ also differed in every respect from ‘statics’, and ‘dynamics’ was at the very heart of how economic theorising ought to look like. He wanted to make economics an evolutionary science: economic evolution was interpreted by Veblen as a dynamic process of cumulative causation, changing the economic system continuously from within. By contrast, a static theory or, as he called it, a ‘taxonomic’ approach, was not part of evolutionary economics (Veblen 1898, 1899).

Both Schumpeter and Veblen are considered pioneers of evolutionary economics. Different aspects of Schumpeter's ideas on dynamic economic systems are discussed for instance in Andersen (2006, 2009), Kurz (2012), Kurz and Sturn (2012), Nelson and Winter (1982), Shionoya (1997), and Swedberg (1991, 2013). Veblen's view on economic evolution and related issues are examined by Hodgson (2008), Latsis (2010), and Rutherford (1994, 2011) among others. This paper seeks to explain what Schumpeter and Veblen meant when talking about *economic evolution*. Based on a study of *the dichotomy between statics and dynamics,* it endeavours to contribute to a better understanding on their ideas of how economics could become an evolutionary science and on their contributions to evolutionary economic theorising. Efforts have been made to combine Schumpeter's respectively Veblen's ideas on dynamic economic systems and on economic evolution with those of other authors: for instance, Michaelides and Milios (2005) study the influence of Hilferding on Schumpeter, while Edgell and Townshend (1993) compare the works of Marx and Veblen in this respect. However, to the best of our knowledge so far no effort has been made to combine the perspectives of Schumpeter *and* Veblen on this topic within a single piece of work. Therefore this paper is a first step towards unifying these historical roots of evolutionary economics. It is shown how the two economists differed in their evolutionary theorising, reflecting also their diverse intellectual and methodological origins.

The paper proceeds as follows: in Section 2, Schumpeter's and Veblen's perceptions of static economic theories are outlined. Their arguments in support of economics becoming an evolutionary science are explained. In Section 3, their paths towards evolutionary economic theorising are discussed in detail. Section 4 provides further insight into their evolutionary economic theorising, through exemplifying their perspectives on the evolution of capitalist economies. Section 5 concludes.

## 2. The relevance of static economics

In *Das Wesen*, Schumpeter summarised the state of the art of pure, static economics at the beginning of the twentieth century. Then the assumption of static behaviour of economic agents was assumed to answer the following questions: ‘Which kind and quantity of commodities do the individual economic agents own? How can this specific distribution of goods and the behaviour of economic agents be understood?’ (*Das Wesen*, pp. 120–1). In a further step he introduced the ‘method of variations’ – comparative static analysis in contemporary terms. With this method, Schumpeter allowed for small changes of parameters (*Das Wesen*, Part IV). The resulting ‘variations are a reaction against the perturbation of equilibrium, leading to a new state of equilibrium’ (*Das Wesen*, p. 451). Yet, if the system is ‘too far’ from equilibrium, static theory was no longer assessed appropriate to explain the properties of the respective system. This led Schumpeter to an extension of (comparative) static economics towards dynamics in *Theorie der wirtschaftlichen Entwicklung* (2006a[1912], henceforth *Theorie*). When Schumpeter clarified his attempt in the revised second German edition of *Theorie*, he suggested ‘a theory of the transition of the economy from a respectively given centre of gravitation to another (“dynamics”), as opposed to the theory of the circular flow itself […] (“statics”)’ (Schumpeter 1952[1926], p. 99). He went even further by arguing that ‘no dynamic equilibrium exists. Economic development by its nature is a disturbance of the existing static equilibrium without any tendency to strive towards this or towards any other equilibrium’ (*Theorie*, p. 489). Also in *Das Wesen*, Schumpeter's assessment of static economics was modest: ‘Dynamic phenomena in our field of research play a greater role than in other exact disciplines. This substantially limits the epistemic value of [static economics]’ (*Das Wesen*, p. 573).^1^ 1 These references to Schumpeter's notion of the state of the art of pure economics at the beginning of the twentieth century resemble Veblen's perception of static economics as discussed in more detail below. However, when Schumpeter in 1926 in the second German edition of *Theorie* wrote about ‘statics’, he perceived neither of a rigorous definition of this term nor of the term ‘dynamics’. Only after reading Frisch's 1929 article^2^ 2 A shortened English translation of Frisch's 1929 article *Statikk og dynamikk i den økonomiske teori* was published in 1992 as *Statics and Dynamics in Economic Theory* in Structural Change and Economic Dynamics. on these issues, in the preface to the English edition of *Theory of Economic Development* (2012[1934], p. xiii) he noted that ‘I at first used the terms “statics” and “dynamics” [for the theory of equilibrium and for the business cycle theory], but have now (in deference to Prof. Frisch) definitely ceased to use them in this sense’. This mere change of words in Schumpeter's writings was highlighted by Hagemann (2003, p. 56): ‘[I]n contrast to the 1926 German edition in the 1934 English edition the terms “statics” and “dynamics” are fully replaced by the concepts of the “circular flow” and “economic development”’.^3^ 3 Whenever ‘statics’ or ‘dynamics’ is mentioned in this paper associated with Schumpeter, they are synonymous to his notions of the ‘circular flow’ and of ‘economic development’, respectively. In this sense Schumpeter followed Machlup's (1959) advice to stop using the terms ‘statics’ and ‘dynamics’ and to replace them by more meaningful concepts. Also the term ‘economic development’ finally passed through a transformation when Schumpeter later on in *Business Cycles* (1964)[1939] and in *Capitalism, Socialism, and Democracy* (2010[1942], henceforth *Capitalism*) used the term ‘economic evolution’ as an equivalent to the term ‘economic development’.

Already at the end of the nineteenth century Veblen in his article *Why is economics not an evolutionary science?* (1898) advocated evolutionary theorising in economics. He opted for a reorientation of economic methodology: ‘[T]he science stands in need of rehabilitation. [… It] is helplessly behind the times, and unable to handle its subject-matter in a way to entitle it to standing as a modern science’ (1898, p. 373). Veblen in this context was concerned with the uncovering of causal relations. He directly looked for dynamic economic theories for which he claimed that the past state of affairs influences the present one, and this occurs as a continuing sequence. Such ‘an evolutionary economics must be the theory of a process of cultural growth […], a theory of a cumulative sequence of economic institutions stated in terms of the process itself’ (1898, p. 393). Veblen did not only concentrate on the necessity of understanding economic systems as evolutionary systems but he was also critical to those economists who followed different lines. According to Veblen, a modern evolutionary economic theory ought to be non-taxonomic and there was no longer a lack of a dynamic perspective or too much of a ‘constraining normality’ (1898, p. 379). Economics up to his time had not become an evolutionary science, since ‘it is this facile recourse to inscrutable figures of speech as the ultimate terms of the theory’ (1898, p. 383).

Against an extension of pure, static economics (i.e. pre-evolutionary economics) towards comparative statics and towards the investigation of equilibrium paths, as first pursued by Schumpeter, Veblen asserted that then observed changes could only be interpreted as a deviation from the normal state (i.e. the static equilibrium) or as ‘disturbing factors’. Instead, for economics to become evolutionary, the so-called disturbing factors were themselves required to take centre stage. Veblen argued that if economics were to be evolutionary, there would be no room for controlling principles such as the concept of equilibrium, since these controlling principles restrict from taking on a dynamic perspective. He considered thus pure static economics as ‘a body of maxims for the conduct of business and a polemical discussion of disputed points of policy’ (1898, p. 384).

Apart from this critique and from their aim to clarify the dichotomy between statics and dynamics, both Veblen and Schumpeter also observed efforts made by their contemporaries and predecessors to push economics towards a dynamic, evolutionary theory. Schumpeter for example referred to John Stuart Mill's *Principles of Political Economy* (1848), in which the first three books are concerned with statics, while the fourth book explicitly deals with dynamics:

All this, however, has only put us in possession of the economical laws of a stationary and unchanging society. We have still to consider the economical condition of mankind as liable to change […] thereby adding a theory of motion to our theory of equilibrium – the Dynamics of political economy to the Statics. (Mill 1848, Book IV, Chapter I; quoted in Schumpeter 1952[1926], p. 85)

Also Veblen honoured Mill for being focused on the explanation of dynamic processes:

J. S. Mill's doctrines of production, distribution, and exchange, are a theory of certain economic processes and […] he deals in a consistent and effective fashion with the sequences of fact that make up his subject matter. (Veblen 1898, p. 375)

Even though Veblen traced dynamic elements in the earlier history of political economy, he saw its failure to turn into an evolutionary science rooted in its concern with explaining every economic phenomenon in terms of some ‘natural law’ (1898, p. 378). With contemporary works he discerned the same failure. Veblen (1908, 1909) in this context referred to John Bates Clark's distinction between statics and dynamics, the latter advocating that

static laws are […] real laws. […] We study them separately, in order that we may understand one part of what goes on in dynamic society. [… I]t is necessary to study the forces of progress. To influences that would act if society were in a stationary state, we must add those which act only as society is thrown into a condition of movement and disturbance. This will give us a science of Social Economic Dynamics. (Clark 1898, p. 10)

Veblen rejected the alleged complementarity of static and dynamic theorising, since this blurred what dynamics and evolution were all about. Clark's avenue towards a dynamic theory he therefore considered as a dead end:

For all their use of the term ‘dynamic’, neither Mr. Clark nor any of his associates in this line of research have yet contributed anything at all appreciable to a *theory of genesis, growth, sequence, change, process*, or the like, in economic life. They have had something to say as to the bearing which given economic changes, accepted as premises, may have on valuation, and so on distribution; but as to the causes of change or the unfolding sequence of the phenomena of economic life they have had nothing to say hitherto; nor can they since their *theory is not drawn in causal terms* […]. (Veblen 1909, pp. 620–1, italics added)

In a similar vein, Schumpeter remarked on Clark's approach (Schumpeter 1910). He credited Clark with having dealt with the problem of economic dynamics: ‘An attempt to provide a theory of economic development and hence to extend the limitations of theoretical economics towards the frontiers of economic life, was carried out only by Clark’ (1910, p. 961). However, after reflecting on Clark's theory, Schumpeter concluded that it is ‘doubtful whether this is a satisfying theory of economic development’ (1910, p. 962). Schumpeter was not convinced by Clark's approach due to the predominance of static considerations in an allegedly dynamic economic theory. As for the case of Clark, Schumpeter noted in retrospect in *History of Economic Analysis* that

a clear distinction between stationary and evolutionary states [was made by Clark]. He identified this indeed with the distinction between statics and dynamics. But this did not greatly matter. He saw the essential points involved in constructing the model of a stationary state and he created, for the purpose of describing its properties, the concept of Synchronization.^4^ 4 Synchronisation means that ‘the flow of consumers’ goods and the flow of productive service are synchronized so that the process works as if society did live on current production’ (Schumpeter 2006b[1954], p. 539). (Schumpeter 2006b[1954], p. 835)

Further evidence for a change of economic method towards an evolutionary perspective was found also in other parts of the history of political economy. These efforts – according to Veblen – were made by the Austrian School and by Marginalists. Yet, similar to his assessment of Clark's approach Veblen did not consider them as successful: ‘[T]he Austrians have on the whole showed themselves unable to break with the classical tradition that economics is a taxonomic science’ (Veblen 1898, p. 389). Hence, for Veblen too little attention was paid to human action. Behavioural traits were just wrapped under a certain hedonistic stereotype. This behaviour suited some standard case but did not allow studying changing human behaviour in context to economic evolution.

Thus, efforts existed to transform economics into an evolutionary science, which in the view of Schumpeter and Veblen were unsatisfactory. While Schumpeter and Veblen were in agreement concerning the evolutionary character of economic systems and in their general claim for a dynamic economic theorising, they had different explanations of why evolutionary economic theorising had not been successfully implemented yet. Consequently they took different paths to formulate theories accounting for this dynamism. What characterises an evolutionary science for Veblen was that it ought to be ‘placed in antithesis to the taxonomic methods and ideals of the pre-evolutionary days’ (Veblen 1899, p. 123). Veblen thus rejected pure, static economic considerations, whereas Schumpeter took them as a starting point to explain economic evolution: ‘Capitalist reality is first and last a process of change. […But] the question […of] a perfectly equilibrated stationary condition of the economic process is […] *almost, though not quite, irrelevant*’ (*Capitalism*, p. 394, italics added). For Schumpeter pure static economic theorising represented the fundament on which some evolutionary theory could be built on, whereas Veblen regarded it as a cage, from which economics ought to break out.

## 3. Towards an evolutionary perspective in economics

In elaborating on an evolutionary theory of economics, Veblen included aspects from sciences like psychology, anthropology, and biology – which he labelled ‘evolutionary sciences’ (Veblen 1898, p. 374) – into his considerations. He partly relied upon their terminology and methodology. Veblen's inclination to absorb the insights of other evolutionary sciences required economics to be, like ‘[a]ny evolutionary science[, …] a close-knit body of theory. It is a theory of a process, of an unfolding sequence’ (Veblen 1898, p. 375). Hence, from a methodological viewpoint, the ‘principle of cause and effect’ – in the sense of an explanation in terms of inter-temporally dependent and sequential events – ought to take centre stage. According to Veblen, putting the principle of cause and effect into the foreground would imply that ‘[t]here is no ultimate term, and no definitive solution except in terms of further action’ (Veblen 1899, p. 124). Veblen's claim for causality was in contrast to what static economics might afford, which first and foremost aimed at providing an understanding of ‘functional relations’, guaranteeing logical coherence.

A few years after Veblen's effort to base evolutionary economic theorising on other evolutionary sciences, also Schumpeter was keen to push economics towards ‘dynamics’, ‘which is part of economics, but outside of [the static] system’ (*Das Wesen*, p. 614). The young Schumpeter took pains to distinguish pure economics from other sciences: ‘Even if comparing two sciences calls forth many concerns, nevertheless it cannot be denied that it adds to the understanding of their nature’ (*Das Wesen*, p. 536). This was exemplified with respect to biology, which was capable ‘[of] scrutiniz[ing] the essence e.g. of economic activity, and it figures out human motivations, something economists cannot do’ (*Das Wesen*, p. 538). Schumpeter still supported this argument about 30 years later, when he wrote that biology provided input for developing an evolutionary economics, for example ‘such a thing as social and economic Darwinism’ (Schumpeter 2006b[1954], p. 25). With respect to the field of psychology, such a contribution to economics was denied in his early writings: ‘[N]o methodological or tangible relation between economics and psychology exists to which we have to refer in order to arrive at our results’ (*Das Wesen*, p. 544). In his later writings, this extreme viewpoint underwent qualification: ‘[I]t is necessary to glance occasionally at the developments in the field of professional psychology’ (Schumpeter 2006b[1954], p. 25). Schumpeter also had a more differentiated point of view concerning the relevance of anthropology and related disciplines for economics. He was clear about their importance in accounting for dynamic aspects in economic theorising, since ‘[f]actual reports and theories about actual events […] are of fundamental importance for the theoretical economists as soon as he leaves the narrow limits of his exact system’ (*Das Wesen*, p. 552). It was only pure and static economics for which he neglected any value of these fields of research ‘[which] are of little use – if they are of any use’ (*Das Wesen*, p. 553). In contrast to Veblen, who promoted an interdisciplinary perspective in order to understand economic evolution, Schumpeter proposed a more ‘intra-disciplinary’ perspective of four different fields of research:

What distinguishes the ‘scientific’ economist from all the other people who think, talk, and write about economic topics is a command of techniques that we class under three heads: *history, statistics*, and ‘*theory*.’ The three together make up what we shall call *Economic Analysis*. […It is] useful […] to introduce a fourth fundamental field to complement the three others […]: the field that we shall call Economic Sociology (Wirtschaftssoziologie). [… E]conomic analysis deals with the questions how people behave at any time and what the economic effects are they produce by so behaving; economic sociology deals with the question how they came to behave as they do. (Schumpeter 2006b[1954], pp. 10, 19, italics added)

It was thus considered important by Schumpeter to acknowledge the mutual dependence of economic and sociological research, resembling Max Weber's focus on social economics (2006b[1954], p. 19).^5^ 5 Schumpeter mentioned his esteem for Weber's efforts in this respect in his History of Economic Analysis (2006b, p. 19). Indeed it seems that Weber, – who invited Schumpeter to write about *Epochen der Dogmen- und Methodengeschichte* in his *Grundriss der Sozialökonomik* (Weber 1922) – provided concepts which Schumpeter adopted for his economic theory (see MacDonald 1965 for a comparison of Schumpeter and Weber). Also Veblen remarked on the importance to include sociological aspects and particularly stressed the connection between social conditions and economic behaviour in explaining his evolutionary ideas: ‘There is the economic life process still in great measure awaiting theoretical formulation’ (1898, p. 387). Thus, an evolutionary economics had to turn its back on hedonism and focus instead on individual behavioural traits, since ‘it is the human agent that changes’ (1898, p. 387). Veblen argued that concentrating more on sociology and aspects of behavioural psychology in evolutionary economic theorising would also allow the application of the principle of cause and effect in a more stringent way and hence manifest the perception of economic processes as being characterised by cumulative change: ‘All economic change is a change in the economic community – a change in the community's methods of turning material things to account. The change is always in the last resort a change in habits of thought’ (1898, p. 391). Veblen's claim for economics to deal more intensely with behavioural traits was reinforced in *The Limitations of Marginal Utility* (1909):

In so far as modern science inquires into the phenomena of life, whether inanimate, brute or human, it is occupied about questions of genesis and cumulative change, and it converges upon a theoretical formulation in the shape of life-history drawn in causal terms. In so far as it is a science in the current sense of the term, any science, such as economics, which has to do with human conduct, becomes a genetic inquiry into the human scheme of life; and where, as in economics, the subject of inquiry is the conduct of man in his dealings with the material means of life, the science is necessarily an inquiry into the life-history of material civilization, on a more or less extended or restricted plan. (1909, pp. 627–8)

In *The Instinct of Workmanship and the State of the Industrial Arts* (Veblen 1918[1914], henceforth *Instinct*) Veblen substantiated his ideas on evolutionary economics and embedded the description of dynamic processes into a detailed treatise on human behaviour. He considered the understanding of individual behavioural traits on the micro-level as pivotal to gain knowledge of evolutionary phenomena on a more aggregate level. Veblen described individual behaviour as being to some degree ‘tropismatic’ but also ‘instinctive’. Contrary to tropismatic behaviour, instinctive action ‘involves consciousness and adaption to an end aimed at’ (*Instinct*, p. 4) and was therefore considered as purposeful and intentionally motivated. Veblen also distinguished between stable and changing patterns of individual behaviour, which allowed him to leave behind the hedonistic stereotypes studied in taxonomic, pre-evolutionary sciences.

Schumpeter in a similar way based his theories on what he called ‘methodological individualism’,^6^ 6 Agassi (1960, 1975) introduces next to ‘methodological individualism’ and ‘methodological holism’ the term ‘institutional individualism’ as a further methodological axis to close the gap between the former two. This distinction between methodological individualism, methodological holism, and institutional individualism was further discussed in Rutherford (1994), who refers to both Veblen and Schumpeter as sharing elements of institutional individualism in their method. which ‘only means that one ought to start with the actions of individuals to describe economic processes’ (*Das Wesen*, p. 90). For pure, static economics Schumpeter derived the equilibrium conditions from individual exchange relations of hedonistic agents, but to understand economic evolution he introduced a second type of idealised agent with a different kind of behaviour – the ‘entrepreneur’:

In contrast to the ‘economic man,’ who carefully calculates marginal costs and revenues of alternative courses of action on the basis of known data, the entrepreneur must be a man of ‘vision,’ of daring, willing to take chances, to strike out, largely on the basis of intuition, on courses of action in direct opposition to the circular flow. (Schumpeter 2012[1934], p. xxi)

Schumpeter considered the entrepreneur's behaviour as being the essential type of individual behaviour driving economic evolution. His motives are of a sociological and economic nature and manifest in his intention to create something new and to break with old traditions related to the functioning of the economic system in the circular flow. As regards this kind of innovative behaviour, Veblen similar to Schumpeter's entrepreneur introduced the ‘instinct of workmanship’ (i.e. ‘sense of workmanship’) paired with ‘idle curiosity’ as individual attributes driving economic evolution. Yet, for Veblen these individual attributes did not take such a significant role for economic evolution as Schumpeter assigned to the entrepreneur. Nonetheless, Veblen argued that the instinct of workmanship ‘occupies the interest with practical expedients, ways and means, devices and contrivances of efficiency and economy, proficiency, creative work and technological mastery of facts’ (*Instinct*, p. 33).

In advancing towards an evolutionary theory, Schumpeter also considered the feedback of social conditions onto individual behaviour as an essential element. Already at the beginning of his career he stated ‘that social influences determine individual actions’ (*Das Wesen*, p. 93). This idea was carried over to his later writings. In a comment on Marx he wrote:

The economic interpretation of history does not mean that men are […] wholly […] actuated by economic motives. On the contrary, the explanation of the role and mechanism of non-economic motives and the analysis of the way in which social reality mirrors itself in the individual psyches is an essential element of [Marx's] theory and one of its most significant contributions. (*Capitalism*, p. 10)

A similar idea of Veblen can be found in *Instinct*. There he argued that cultural, institutional, and organisational conditions feed back on the individual level. Individual behaviour was considered as being socially co-determined and guided by socio-economic conditions. Even though socio-economic conditions established specific stable habits of thought, routines, and norms in a society, these were not seen as being of a persistent character but were expected to undergo change, reflecting in ‘new habits of work and of thought in the community, and so [these changes] continually instill new principles of conduct’ (*Instinct*, p. 17).

Thus, evolution and consecutive change in the socio-economic environment were accentuated in Veblen's and Schumpeter's evolutionary perspectives. For them this happened on the individual as well as on the level of society as a whole, showing in institutional and organisational innovations or in ‘unremitting changes and adaptions that go forward in the scheme of institutions, legal and customary’ (*Instinct*, p. 17). Additionally, economic evolution was considered to take place in the material environment, reflecting what Schumpeter in *Theorie* described as process and product innovations:

[Development] covers the following five cases: (1) The introduction of a new good […] or of a new quality of a good. (2) The introduction of a new method of production […]. (3) The opening of a new market […]. (4) The conquest of a new source of supply of raw materials or half-manufactured goods[…]. (5) The carrying out of the new organisation of any industry […]. (Schumpeter 2012[1934], p. 66)

According to Schumpeter, the driving force of economic evolution is therefore the emergence of innovations: ‘The changes in the economic process brought about by innovation, together with all their effects, and the response to them by the economic system, we shall designate by the term Economic Evolution’ (Schumpeter 1964[1939], p. 83). In a similar vein, Veblen argued:

The complex of technological ways and means grows by increments that come into the scheme by way of improvements, innovations, expedients designed to facilitate, abridge or enhance the work to be done. Any such innovation that fits workably into the technological scheme, and that in any appreciable degree accelerates the pace of that scheme at any point, will presently make its way into general and imperative use, regardless of whether its net ulterior effect is an increase or a diminution of material comfort or industrial efficiency. (*Instinct*, p. 314)

Neither for Veblen nor for Schumpeter the evolution of technologies, institutions, and organisations on the level of society as a whole as well as changes in individual behaviour was considered independent from each other. These types of dynamic processes appeared as cumulative causation and were strongly tied together: ‘When a step in development has been taken, this step itself constitutes a change of situation which requires a new adaption; it becomes the point of departure for a new step in the adjustment’ (Veblen 1922[1899], p. 191). According to Veblen, it were thus the processes of continuous and complementary self-reinforcing change, both in the socio-economic environment and in the material environment, which constituted the basis for the evolution of economic systems: ‘Technological change in Veblen's work occurs at a pace and in a direction affected by the existing institutional framework as manifested in the habitual ways of thinking’ (Rutherford 1994, p. 38). For Veblen ‘[t]echnology has institutional consequences by altering material circumstances and the methods, patterns, and habits of life and thought of individuals’ (1994, p. 39). This materialistic view, which roots in the Marxian notion of material historicism,^7^ 7 Marx outlined his ideas of how history evolves based on economic circumstances throughout his writings and made these explicit in the *Communist Manifesto*: ‘The basic thought running through the Manifesto–that economic production and the structure of society of every historical epoch necessarily arising therefrom constitute the foundation for the political end intellectual history of that epoch’ (Marx and Engels 1967[1848], p. 57). was also shared by Schumpeter, who assessed economic sociology as pivotal to understand society: ‘To use a felicitous phrase: economic analysis deals with the questions how people behave at any time and what the economic effects are they produce by so behaving; economic sociology deals with the question how they came to behave as they do’ (Schumpeter 2006b[1954], p. 19). And it is ‘human behavior which we assume either in general or for certain social situations but not for others’ (2006b[1954], p. 18). Schumpeter substantiated his ideas of the consequences of technical and economic circumstances on human behaviour in *Capitalism* (pp. 122–3): ‘Now the rational attitude presumably forced itself on the human mind from economic necessity; it is the everyday economic task to which we owe our elementary training in rational thought and behaviour’.

## 4. Capitalist dynamics under an evolutionary perspective

Following their assessment of the interdependence between changes in the material and the socio-economic environment as well as the connection to individual behaviour, Veblen and Schumpeter devoted considerable parts of their scientific work on developing ideas of how meaningful evolutionary economic theorising ought to look like. In this respect, they were *inter alia* concerned with the following interrelated but distinct questions: *Which factors do characterise modern capitalist economies and how does the capitalist system behave over time? Are there processes leading to a transformation of the capitalist order over time?* Hence, the study of capitalist economies in both Veblen and Schumpeter was not only limited to understand the system-inherent forces driving the evolution of capitalist societies (Section 4.1) but also to grasp the consequential social transition of capitalist economies towards some alternative economic order (Section 4.2).

### 4.1 Processes of change under the capitalist system

For Schumpeter the pivotal point for understanding the complex processes driving capitalist systems from within was the observation that

in dealing with capitalism we are dealing with an evolutionary process […] due to the fact that economic life goes on in a social and natural environment which changes and by its changes alters the data of economic action. (*Capitalism*, p. 72)

The need to simultaneously account for changes in the socio-economic, material, and natural environment and therefore study economic evolution as dynamic process in which these environments co-evolve was emphasised by Veblen when he studied the evolution of capitalist economies *inter alia* in *The Theory of Business Enterprise* (1904, henceforth *Business Enterprise*). In concentrating on the evolution of western civilisations (especially of the United States), Veblen discussed the transformation from the money economy of earlier times to the credit economy under the capitalist economic system. When he described the attributes characterising capitalist economies or as he called it, the ‘modern industrial system’ (*Business Enterprise*, p. 1), he time and again stressed the complexity and interdependency of processes prevailing within the system: ‘[T]he modern industrial system is a concatenation of processes’ (*Business Enterprise*, p. 8). As relevant processes inherent to the evolution of capitalist societies Veblen identified the ‘machine process’ and the ‘business enterprise’. Concerning the former, this was considered to comprise the production system of an economy, which with the evolution of capitalist economies had become largely standardised and institutionalised, reflected in an ‘accuracy in point of time and sequence, in the proper inclusion and exclusion of forces affecting the outcome, in the magnitude of the various physical characteristics’ (*Business Enterprise*, p. 3). Regarding the business enterprise, Veblen subsumed the organisation of the machine process through business principles and other social institutions. One of the most fundamental business principles in capitalist societies, according to Veblen, was the institution of free ownership:

As the machine process conditions the growth and scope of industry, and as its discipline inculcates habits of thought suitable to the industrial technology, so the *exigencies of ownership* condition the growth and aims of business, and the discipline of ownership and its management inculcates views and principles (habits of thought) suitable to the work of business traffic. (*Business Enterprise*, p. 23, italics added)

Connecting in a further step changes in the machine process and in the business enterprise to individual action, Veblen integrated elements of behavioural psychology and introduced another stereotype of individual behaviour – the modern ‘businessman’, whose motives of action lay in a rather anxious and historically unprecedented striving for ‘investment for profit’ (*Business Enterprise*, p. 2).^8^ 8 This stereotype included bankers, managers, brokers as opposed to the engineers and the working class and was considered to behave as both instinctive and non-tropismatic. Veblen assigned an overwhelming and central position to the businessman, who is the

controlling force of industry […] because through the mechanism of investments and markets, he controls the plants and processes, and these set the pace and determine the direction of movement for the rest […]. [H]e is the only large self-directing economic factor [… and] the large business man controls the exigencies of life under which the community lives. Hence, upon him and his fortunes centres the abiding interest of civilized mankind. (*Business Enterprise*, p. 2)

In Veblen's point of view, businessmen did not act in routines but were apt to change the direction of the enterprise. To grasp this aspect of capitalism Schumpeter referred to the entrepreneur, who initiates ‘revolutions’ which ‘periodically reshape the existing structure of the industry by introducing new methods of production […]; new forms of organization […]; new sources of supply […]; new trade routes and markets to sell’ (*Capitalism*, p. 59). Yet, Schumpeter's entrepreneur behaved differently to Veblen's businessman in such a way as that the latter

does not go creatively into the work of perfecting mechanical processes and turning the means at hand to new and or larger uses. That is the work of the men who have in their hand the devising and oversight of mechanical processes. The men in industry must first create the mechanical possibility of such new and more efficient methods and correlations, before the business man sees the chance, makes the necessary business arrangements, and gives general directions that the contemplated industrial advance shall go into effect. (*Business Enterprise*, p. 13)

Under competitive conditions and with changes in the business enterprise in capitalist societies, the businessman's profit-seeking actions had led to a rapid increase in significance of the credit market compared to the older money economy. This reflected in steady credit creation and in ‘capitalization [which] is comprised the usufruct of whatever credit extension the given business concern's industrial equipment and good-will will support’ (*Business Enterprise*, p. 40).^9^ 9 At the same time, Veblen considered the businessman also as saboteurs of capitalism, since their behaviour induces non-competitive market situations: ‘“Cutthroat” competition that is to say, free competitive selling, can be done away by “pooling the interests” of the competitors, so soon as all or an effective majority of the business concerns which are rivals in the market combine and place their business management under one directive head. When this is done, by whatever method, selling of goods or services at competitively varying prices is replaced by collective selling (“collective bargaining”) at prices fixed on the basis of “what the traffic will bear”’ (*Business Enterprise*, p. 80). Veblen stressed that extensive credit creation and the related process of capitalisation were an outcome of the growing significance of the business enterprise in the evolution of capitalist societies. Furthermore, this changing function of the credit market resulted in

[c]rises, depressions, hard times, dull times, brisk times, periods of speculative advance, “eras of prosperity,” [which] are primarily phenomena of business; they are, in their origin and primary incidence, phenomena of price disturbance, either of decline or advance. It is only secondarily, through the mediation of business traffic, that these matters involve the industrial process or the livelihood of the community. They affect industry because industry is managed on a business footing, in terms of price and for the sake of profits. (*Business Enterprise*, p. 60)

Also Schumpeter included monetary considerations into his analysis of the processes driving capitalist evolution and similar to Veblen he emphasised that the establishment of market situations, which deviate from competitive markets – monopoly and oligopoly – are inherent to the evolution of capitalist systems. Non-competitive market situations were thus key issues in Schumpeter's and Veblen's writings on capitalist evolution. Schumpeter's starting point was the ‘[c]ommercial society […] defined by […] private property in means of production and regulation of the productive process by private contract’ (*Capitalism*, p. 149).^10^ 10 To avoid misunderstandings for those who had perfect competition in mind, Schumpeter stressed that ‘the modern standard of life of the masses evolved during the period of relatively unfettered “big business”’ (*Capitalism*, p. 71). Describing the dynamic processes underlying capitalist societies hence asks for a theory looking at oligopolistic and monopolistic behaviour:

As soon as we go into details and inquire into the individual items in which progress was most conspicuous, the trail leads not to the doors of those firms that work under conditions of comparatively free competition but precisely to the doors of the large concerns (*Capitalism*, p. 72).

Changes in business structures due to organisational and institutional innovations, according to Schumpeter, feed back on individual behaviour:

The impact of new things–new technologies for instance–on the existing structure of an industry considerably reduces the long-run scope and importance of practices that aim […] at conserving established positions and at maximizing the profits accruing from them. (*Capitalism*, p. 77)

These changes which hit the system by means of innovations were considered as

not strictly incessant; they occur in discrete rushes which are separated from each other by spans of comparative quiet. The process as a whole works incessantly however, in the sense that there always is either revolution or absorption of the results of revolution, both together forming what are known as business cycles. (*Capitalism*, p. 395)

If basic innovations were introduced by chance they would co-determine economic activity throughout. Schumpeter deemed their appearance and effects as unpredictable: ‘[T]here is no point in appraising the performance of [the new] process *ex visu* of a given point of time’ (*Capitalism*, p. 73). There only existed one regularity in economic evolution which Schumpeter already in *Theorie* described in detail, namely the occurrence of business cycles as a consequence of innovative behaviour. This sequence of up- and downswings he explained as follows:

[T]he boom and the depression begins after the passage of the time which must elapse before the products of the new enterprises can appear on the market. And a new boom succeeds the depression when the process of resorption of the innovations is ended. (Schumpeter 2012[1934], p. 213)

According to Schumpeter, business cycles, which he discussed *inter alia* in chapter 6 of *Theorie,* were an outcome of swarms of innovators hunting for profits. This behaviour eventually would lead to overproduction and to a subsequent recession. He summed up this process of economic change from within as a process where new products and processes emerged alongside old ones and finally replaced them. His theory hence explains:

The effect of the appearance of new enterprises en masse upon the old firms and upon the established economic situation […]. [A]s a rule the new does not grow out of the old but appears alongside of it and eliminates it competitively, is so to change all the conditions that a special process of adaptation becomes necessary. (2012[1934], p. 216)

Since Schumpeter observed that ‘[i]t is a fact that the economic system does not move along continually and smoothly’ (2012[1934], p. 216), he expanded his ideas on the emergence of business cycles, differentiating between crises triggered by non-economic reasons – such as for instance wars and meteorological circumstances (2012[1934], p. 220) – as well as crises caused by pure economic phenomena. Only the latter were of interest for Schumpeter in chapter 6 of *Theorie*, and he claimed that business cycles emerged ‘exclusively *because the new combinations are not, as one would expect according to general principles of probability, evenly distributed through time* […] but *appear, if at all, discontinuously in groups or swarms*’ (2012[1934], p. 223). Even more, swarms of innovators emerge ‘[e]xclusively because the appearance of one or a few entrepreneurs facilitates the appearance of others, and these the appearance of more, in ever-increasing numbers’ (2012[1934], p. 228). This in turn would lead to decreasing profits and finally to some mismatch of effective demand. Nevertheless, ‘[a]lthough it stands to reason that the process of adjustment and resorption which makes up the period of depression causes discomfort’ (2012[1934], p. 241), Schumpeter highlighted two important aspects of these kinds of depressions characterising capitalist economies: first, they led to some new state of equilibrium and second,

it fulfils what the boom promised. And this effect is lasting, while the phenomena felt to be unpleasant are temporary. The stream of goods is enriched, production is partly reorganised, costs of production are diminished, and what at first appears as entrepreneurial profit finally increases the permanent real incomes of other classes. (2012[1934], p. 245)

Similar to Schumpeter's point of view, according to Veblen capitalist economies were bound to deep cyclical movements inherent to the system, which resulted from a conflict between changes in the business enterprise and in the machine process:

The true, or what may be called the normal, crises, depressions, and exaltations in the business world are not the result of accidents, such as the failure of a crop. They come in the regular course of business. […] In the recent past, since depression and exaltation have been normal features of the situation, every strongly marked period of exaltation (prosperity) has had its attendant period of depression (*Business Enterprise*, p. 61, italics added).

### 4.2 From capitalism to socialism

As a consequence of the dynamic processes of change driving the evolution of capitalist societies, both Veblen and Schumpeter projected a long-term transformation of capitalist societies to some alternative economic order. According to both of them, this manifested in terms of an ideological shift. Veblen assessed the institutionalisation and standardisation of the machine process under capitalist economies as being decisive for this transformation:

The machine process pervades the modern life and dominates it in a mechanical sense. Its dominance is seen in the enforcement of precise mechanical measurements and adjustment and the reduction of all manner of things, purposes and acts, necessities, conveniences, and amenities of life, to standard units. (*Business Enterprise*, p. 104)

The institutionalisation of the machine process and its sphere of action in modern life, Veblen argued, led to changes of individual behaviour: ‘The machine throws out anthropomorphic habits of thought. It compels the adaptation of the workman to his work, rather than the adaptation of the work to the workman’ (*Business Enterprise*, p. 105). Thus, one outcome of the growing importance of the machine process in the capitalist system was that ‘[w]herever the machine process extends, it sets the pace for the workmen, great and small’ (*Business Enterprise*, p. 104) and this would lead to ‘a standardization of the workman's intellectual life in terms of mechanical process’ (*Business Enterprise*, p. 104). Even though Veblen considered changes in the machine process (i.e. technological change) as having induced great improvements in the material conditions of societies, he saw a conflict between this and changes in the business enterprise. For Veblen, the predominance of the machine process related to its routinisation and standardisation was an origin of class struggle between the class of the businessmen and the working class:

[T]he fact still is apparent that the everyday life of those classes which are engaged in business differs materially in the respect cited from the life of the classes engaged in industry proper. There is an appreciable and widening difference between the habits of life of the two classes […]. So that the two classes come to have an increasing difficulty in understanding one another and appreciating one another's convictions, ideals, capacities, and shortcomings. (*Business Enterprise*, p. 107)

Changes in habits of thought of the working class induced by a growing material welfare in capitalist societies Veblen deemed a dead end to the evolution of capitalist societies. These changes would result in a successive disruption of natural rights and an excavation of the institution of free ownership. Furthermore, the social conflict between the working class and the class of the businessmen would be fuelled, since

[t]he conditions of life imposed upon the working population by the machine industry discourage thrift. But after allowance has been made for this almost physical restraint upon the acquisition of property by the working classes, something is apparently left over, to be ascribed to the moral effect of the machine technology. The industrial classes appear to be losing the instinct of individual ownership. The acquisition of property is ceasing to appeal to them as a natural, self-evident source of comfort and strength. The natural right of property no longer means so much to them as it once did. (*Business Enterprise*, p. 109)

Emphasising again the interdependence between the machine process and the business enterprise Veblen argued that ‘in the long run [the business enterprise cannot] get along with the [development of the] machine process’ (*Business Enterprise*, p. 126). Veblen projected thus a transition from the capitalist system, based on ‘certain natural rights, particularly, those of property and free contract’ (*Business Enterprise*, p. 112), to some kind of socialist order with a trend to abandon property rights and to monopolisation, going hand in hand with a gradual expulsion of the business enterprise.

Another source intimately connected to the transformation of capitalist societies towards some socialist order in terms of an ideological shift Veblen saw in the growing influence of trade unions: ‘Trade unionism denies individual freedom of contract to the workman, as well as free discretion to the employer to carry on his business as may suit his own ends’ (*Business Enterprise*, p. 110). Thus, again changes in habits of thought of the working class and especially of those workers engaged in trade unionism fostered this transformation:

When distrust of business principles rises to such a pitch as to become intolerant of all pecuniary institutions […], it is spoken of as “socialism” or “anarchism.” […]. The immediate point of danger in the socialistic disaffection is a growing disloyalty to the natural−rights institution of property […]. The classes affected with socialistic vagaries protest against the existing economic organization, but they are not necessarily averse to a somewhat rigorous economic organization on new lines of their own choosing. (*Business Enterprise*, p. 112)

Also Schumpeter described capitalist evolution as leading to tendencies of socialism: it is the struggle for survival of business units not on the basis of perfect or free competition but due to monopolistic competition. Schumpeter thus considered perfect competition as an unstable situation in the evolution of capitalist economies. As discussed by Michaelides and Milios (2005), in Schumpeter's notion competitive markets over time were counteracting innovations in capitalist societies and he therefore considered them as not favouring economic evolution. Contrary to Veblen's arguments in terms of behavioural psychology, for Schumpeter this was due to sociological conditions, as perfect competition leads to the excavation of profits related to innovation. According to Schumpeter, this subsequently implied a lack of incentive to innovate and to undertake the risk associated with innovative behaviour. In this context, Schumpeter in *Capitalism* focused on research and development as a routinised and consolidated process to create inventions – only some of which become innovations – and this he regarded as a situation which encourages the establishment of big concerns: ‘Competition will be discouraged by heavy capital requirements or lack of experience, or that means are available to discourage or checkmate it so as to gain the time and space for further developments’ (*Capitalism*, p. 78). As soon as monopolistic tendencies had prevailed, a stagnant situation was incumbent: ‘When there is no perfect competition and when each industrial field is controlled by a few big concerns, these can in various ways fight the threatening attack on their capital structure’ (*Capitalism*, p. 83). Referring to the situation observed after World War I in some European countries, Schumpeter argued that the establishment of trusts and big concerns will – especially among political and intellectual elites – lead to criticism of capitalism, fuelled by social discontent. Political and intellectual elites would protest against inequality and unemployment. And the formation of trade unions would result in an institutional environment, which he considered as harmful to free ownership. Similar to Veblen, Schumpeter saw thus the decline of capitalism as a consequence of the excavation of the institution of free ownership going hand in hand with a suppression of entrepreneurship. Schumpeter projected the decline of capitalism as the result of its own success. This transformation towards some socialist order he considered the final stage of capitalist evolution:

[T]hat the actual and prospective performance of the capitalist system is such […] that its very success undermines the social institutions which protect it, and ‘inevitably’ creates conditions in which it will not be able to live and which strongly point to socialism as the heir apparent. (*Capitalism*, pp. 53, 145)

He understood socialism as ‘an institutional pattern in which the control over means of production and over production itself is vested with a central authority – or, as we may say, in which, as a matter of principle, the economic affairs of society belong to the public and not to the private sphere’ (*Capitalism*, p. 150). Summing up, while the transformation from capitalist societies to some socialist order for Veblen originated from psychological processes, Schumpeter considered it as a result of socio-economic processes.

## 5. Conclusion

Schumpeter as well as Veblen developed and held sophisticated views on the interpretation of ‘dynamics’ and its suitability for uncovering evolutionary phenomena. Using a metaphor, one can say that both climbed the same hill of evolutionary economic theorising from different base camps and with diverse equipment. In their evolutionary theorising, Veblen relied on anthropology, psychology, and biology, whereas Schumpeter was closer to sociology and history. Another demarcation line between these two economists can be found with respect to the appraisal of pure economics. This is well reflected in their references to and attitude towards their contemporaries' and predecessors' works on the dichotomy between statics and dynamics and its relation to pure economic theorising. While Veblen wanted to revolutionise economics and saw pure, static economics as incompatible with an evolutionary economic theorising, Schumpeter chose, so to speak, a more modest way to develop an evolutionary economic theory.

Despite these differences their basic understanding of economic systems shows striking similarities. Both authors approached the field of evolutionary theorising by leaving the field of pure economic theorising. Both of them can be reconciled concerning their notion of evolutionary change arising from within the system. Furthermore, for both of them, economic evolution resolves in a concatenation of different endogenous processes prevailing on different levels: Thus, economic evolution proceeds through various different channels – changes are observed in the material environment and in the socio-economic environment, on the individual level and on the level of society as a whole. Veblen's and Schumpeter's understanding of evolutionary economic systems also resembles in the way they perceived of individual behaviour and changes to it. From a methodological viewpoint, what accounted for both was to introduce changing individual behaviour. While Veblen above all focused on behavioural psychology, Schumpeter predominantly relied on sociological arguments in this respect.

Other similarities between Veblen's and Schumpeter's ideas on how an evolutionary economics ought to look like can be found in their explanations of capitalist evolution: they both did not restrict their evolutionary approaches to the trend of some economic variable. Instead, they included the transformation of society itself, resolved by a concatenation of processes prevailing in capitalist systems. Capitalist societies are characterised by the possibility of free negotiation of contracts and private property. Yet, inherent to the evolution of capitalist societies is the manifestation of monopolistic tendencies, which according to Veblen source from a standardisation and routinisation of the machine process. For Schumpeter, it was the standardisation and routinisation of research and development activities, which suppresses free competition. In the end, the decline of capitalism for both of them induced a transition towards socialism. It is this transition from one economic order to an alternative one, exemplified in this paper by means of the evolution of capitalist societies, which makes the economic theories of Schumpeter and Veblen evolutionary, as compared to allegedly dynamic economic theories which are only concerned with pure economic considerations. They were focused on the economic process embedded in distinct social, material, and cultural environments which continuously change from within. Creative destruction in the Schumpeterian sense, so to speak, is therefore *not* the endpoint. It is the *starting point* of a more comprehensive line of argument including social transition. This might be deemed one of the most distinct elements of Veblen's and Schumpeter's evolutionary economics.
